# Adult Laryngotracheobronchitis in the Setting of a COVID-19 Infection

**DOI:** 10.7759/cureus.68188

**Published:** 2024-08-30

**Authors:** Arjun H.M, Varsha Shinde, Suhrith Bhattaram

**Affiliations:** 1 Emergency Medicine, Dr. D. Y. Patil Medical College, Hospital and Research Centre, Dr. D. Y. Patil Vidyapeeth, Pune, Pune, IND; 2 Emergency Medicine, Maitland Hospital, Newcastle, AUS

**Keywords:** subglottic narrowing, racemic epinephrine, sars-cov-2, covid-19, croup

## Abstract

We describe a case of adult croup in an 18-year-old female caused by the SARS-CoV-2 virus. Her complaints started as lower respiratory tract symptoms that evolved into stridor, barking cough, and dyspnea. The patient was diagnosed with SARS-CoV-2 by reverse transcription-polymerase chain reaction (RT-PCR) testing from a nasopharyngeal swab. The patient received multiple doses of nebulized racemic epinephrine with minimal improvement, and later the patient required mechanical ventilation. Intravenous remdesivir was administered for five days. Multiple doses of dexamethasone were required throughout the course of the illness. Croup in adults secondary to COVID-19 infections appears to be severe and might be poorly responsive to standard treatment protocols.

## Introduction

The COVID-19 pandemic has affected millions of individuals worldwide, causing mortality in both adults and children. Fever, cough, breathlessness, and diarrhea were some of the earliest noted symptoms. As the disease spread, more atypical clinical features and complications were identified, including anosmia, dysgeusia, thromboembolic activity, silent hypoxia, and Kawasaki-like symptoms in children [1]. Only a few cases of SARS-CoV-2-associated croup have been reported across the world, all of them in children [2]. Croup is more common in children aged six months to six years and is typically seen in the late fall and early winter seasons [3]. This case report describes a case of croup in an adult with a SARS-CoV-2 infection that was successfully treated in the emergency department (ED).

## Case presentation

A previously healthy 18-year-old female presented to the ED complaining of guttural respiration, harsh nonproductive cough, fever, muffled voice, and dyspnea. The constitutional symptoms had progressed over the previous three days, with breathlessness and a barking cough developing in the preceding 24 hours. A review revealed a history of well-controlled asthma and contact with COVID-19-positive individuals one week prior.

On arrival, she was febrile (39°C), tachycardic (130/minute), and tachypneic (45/minute), with a blood pressure of 100/70mmHg and oxygen saturation of 92% on room air. A physical examination revealed bilaterally decreased air entry with chest wall retractions. No other significant systemic findings were noted. Her Westley Croup Score was 5, suggesting moderate severity.

The patient was treated with nebulized racemic epinephrine (NRE), intravenous dexamethasone, and intravenous paracetamol. Two doses of NRE were given in the next 40 minutes for persistent stridor and respiratory distress. An hour after the presentation, the patient became drowsy and disoriented as the hypoxia worsened. The patient was intubated with a single-lumen cuffed endotracheal tube of 6.0 and started on mechanical ventilation. Her larynx was inflamed with vocal cord edema, and the pooling of secretions was noted.

**Figure 1:**
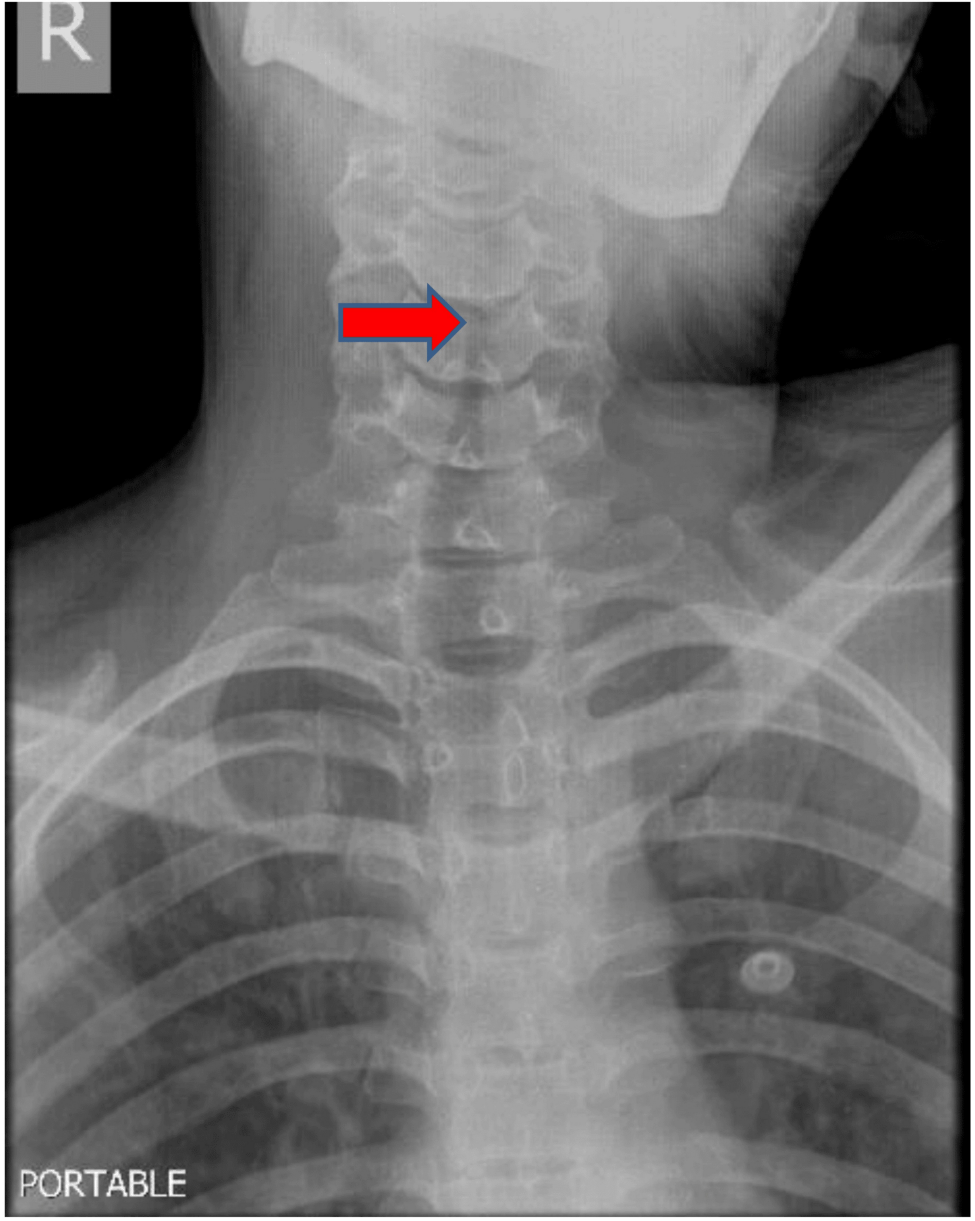
The red arrow shows subglottic narrowing.

Laboratory evaluation revealed leukocytosis (32,000 cells/microliter) with lymphopenia, an unremarkable metabolic panel, and normal urinalysis. Her arterial blood gas was suggestive of type II respiratory failure, with mild metabolic acidosis and elevated lactates. The bedside portable radiography showed subglottic narrowing (Figure 1).

Broad-spectrum antibiotics were initiated, and dexamethasone and epinephrine nebulizations were continued. A positive nasopharyngeal polymerase chain reaction for SARS-CoV-2 was obtained, and the patient was transferred to the COVID-19 isolation ICU for further management. Intravenous remdesivir was administered for five days, and on day five, the patient was extubated. The patient remained asymptomatic two months after the initial admission. 

## Discussion

This case report, to the best of our knowledge, is the first documented evidence of croup in adults with a secondary SARS-CoV-2 infection. Laryngotracheobronchitis, commonly known as croup, is typically a viral disease causing laryngeal and tracheal inflammation. It is commonly seen in the pediatric age group and is rare in adults, because adult airways become larger and more rigid, reducing the susceptibility to the negative pressures of inhalation [4]. Adults and children typically present with high-grade fever, stridor, barking cough, and dyspnea. Croup is commonly caused by parainfluenza type 1, influenza A and B, Respiratory syncytial virus, and adenovirus [5]. Croup is rare in adults, and the pathogenesis remains uncertain [6].

In this described case of adult croup, the patient had stridor at rest and was unresponsive to multiple doses of NRE. She also required administration of multiple doses of dexamethasone, which is an infrequent occurrence in our ED practice. A case series on COVID-19 croup in children has also demonstrated that all three patients had required multiple doses of NRE and additional therapeutic interventions in the form of heliox and non-invasive ventilation (NIV) support [2]. The classical subglottic narrowing “steeple sign” was noted in our patient. This has been demonstrated to be a common radiological presentation, with an incidence of 92% in infected adults but only 26% in pediatric cases [3].

Adult croup is known to be more severe and requires aggressive management, ICU care, and prolonged hospitalization. Although only 15 cases of adult croup have been reported in the literature [4,7,8], the data show that 87% of adults were admitted to the ICU, and nearly half of them required intubation [8]. Treatment in the pediatric group is based on croup severity, but there are no formal recommendations for adult cases. It can be extrapolated that croup in adults secondary to COVID-19 infections can present with severe pathologies and might be poorly responsive to standard treatment protocols. Novel COVID-19 presentations are evolving worldwide, and identifying previously unrecognized symptomatologies is key to developing better therapeutic and quarantine guidelines.

## Conclusions

Our case report identifies a case of COVID-19 croup in an adult patient and also reinforces previous observations that adult croup is more severe, requires far more aggressive management, and might not improve with typical treatment. Further studies are needed before effective therapeutic recommendations can be formulated for the optimal management of COVID-19 croup.
